# Analytical Solution for the Free Vibration Analysis of Delaminated Timoshenko Beams

**DOI:** 10.1155/2014/280256

**Published:** 2014-01-19

**Authors:** Ramazan-Ali Jafari-Talookolaei, Maryam Abedi

**Affiliations:** ^1^Department of Mechanical Engineering, Islamic Azad University, Sari Branch, Mazandaran Province, Sari 48161-194, Iran; ^2^School of Mechanical Engineering, Sharif University of Technology, Tehran 14588-89694, Iran

## Abstract

This work presents a method to find the exact solutions for the free vibration analysis of a delaminated beam based on the Timoshenko type with different boundary conditions. The solutions are obtained by the method of Lagrange multipliers in which the free vibration problem is posed as a constrained variational problem. The Legendre orthogonal polynomials are used as the beam eigenfunctions. Natural frequencies and mode shapes of various Timoshenko beams are presented to demonstrate the efficiency of the methodology.

## 1. Introduction

Delaminations are probably the most frequently occurring damage in beams. Delaminations may originate during fabrication or may be service-induced, such as by impact or fatigue loading. Delaminations not only affect the strength of the structure by contributing to its final failure but also cause a reduction in the stiffness, thus affecting its dynamic characteristics. In particular, delaminations reduce the natural frequency, which may cause resonance if the reduced frequency is close to the working frequency. Therefore, it is necessary to be able to predict the changes in the frequency, as well as the mode shape.

It has been known for many years that the classical Euler-Bernoulli beam theory is able to predict the frequencies of flexural vibration of the lower modes of thin beams with adequate precision. During the past decades, the free vibrations of the delaminated Euler-Bernoulli beams have received considerable attention by many researchers [1–10], but only few publications were devoted to include the effects of shear deformation and rotary inertia for beams [11–13]. Because of the existence of the delamination, the subbeams, especially in a delaminated segment, may no longer remain thin in the beam configuration even though the original undelaminated beam may be thin.

To study the free vibration of an isotropic beam with a through-width delamination, Wang et al. [1] have presented an analytical solution by treating the delaminated beam as four Euler-Bernoulli beams connected at the delamination boundaries. The coupling effect of the longitudinal and flexural motions on the delaminated subbeams was considered in their formulations. It was found that for beams with a short delamination and close to the midplane, the results for the natural frequencies were close to the experimental results. However, according to this study, dramatic interpenetration of the delaminated subbeams was seen that is physically impossible in the case of off midplane delaminations. This is because the delaminated beams were assumed to deform freely without touching each other (known as *free mode*) and thus have different transverse deformation. To avoid this kind of incompatibility, Mujumdar and Suryanarayan [2] proposed a model based on the assumption that the delaminated layers are constrained to have identical transverse deformations (known as *constrained mode*). The *constrained mode* analysis failed to predict the opening in the mode shapes. Free vibrations of delaminated beam-plates with respect to postbuckled referential states have been studied by Yin and Jane [3]. Jane and Chen [4] have considered a homogeneous isotropic beam plate subjected to axial compressive load along two clamped edges with an arbitrary delamination. Based on the continuity conditions of both delaminated tips, the small-amplitude vibration of the delaminated beam plate with respect to a postbuckled state has been studied. An analytical solution to the free vibrations of a beam with two overlapping delaminations has been presented by Della et al. [5, 6]. The delaminated beam has been analyzed as seven interconnected Euler-Bernoulli beams. The *free* and *constrained mode models* have been considered to obtain the lower and upper bounds of the natural frequencies, respectively. Della and Shu [7] have studied analytically the vibration of beams with double delaminations based on the classical beam theory. Later, Della and Shu analyzed the free vibration of delaminated bimaterial [8, 9] beams and beams with two overlapping delaminations in prebuckled states [10]. Both the *free mode* and *constrained mode* analyses have been used in the vibrational study of delaminated beam.

Valoor and Chandrashekhara [11] extended the *constrained mode model* for thick beams to include the effects of the transverse shear deformation and the rotary inertia. However, the *constrained mode* analysis failed to predict the opening in the mode shapes found in the experiments by Shen and Grady [12]. To simulate the “open” and “closed” behaviors between the delaminated surfaces, Luo and Hanagud [13] presented an analytical model based on the Timoshenko beam theory, which uses piecewise-linear springs. The spring stiffness is then set to be equal to zero (0) for the *free mode* and infinity (*∞*) for the *constrained mode*.

In the present paper, a novel analytically approach is proposed for the problem of the free vibrations of a Timoshenko beam without resorting to numerical approximation, in which the orthogonal Legendre polynomials in conjunction with Lagrange multipliers are used. The frequencies and the corresponding mode shapes for common types of boundary conditions are compared extremely well with the available results.

## 2. Problem Formulation 

**Figure 1:**
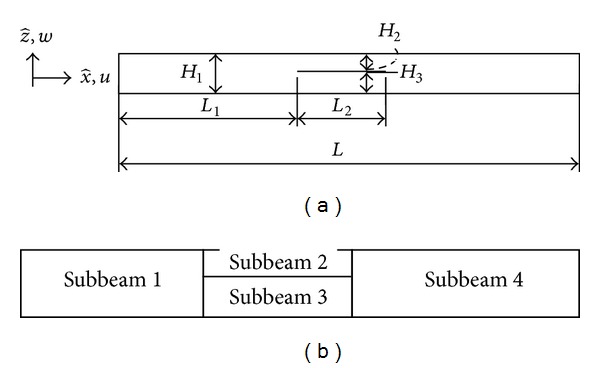
Geometry of a delaminated beam: (a) a beam with a single delamination and (b) four interconnected subbeams.

Consider a straight Timoshenko beam of length *L*, a uniform cross-sectional area *A*, the mass per unit length of *m*, the second moment of area of the cross-section *I*, Young's modulus *E*, and shear modulus *G*. We assume that the beam is made of a homogeneous and isotropic material. As shown in Figure 1(b), after delamination, a representative beam can be viewed as a combination of four beams connected at the delamination boundaries $\hat{x}=L_{1}$ and $\hat{x}=L_{1}+L_{2}$.

In this way, we will have four subbeams of 1 to 4 with lengths and thicknesses of *L*_*i*_ × *H*_*i*_ (*i* = 1 to 4), where *L*_2_ = *L*_3_, *L*_4_ = *L* − *L*_1_ − *L*_2_, *H*_1_ = *H*_4_, and *H*_2_ and *H*_3_ are the thicknesses of subbeams 2 and 3, respectively. Because of the existence of the delamination, the subbeams, especially in a delaminated segment, may no longer remain thin in the beam configuration even though the original undelaminated beam may be thin.

To simulate the “open” and “closed” behaviors between the delaminated surfaces, we use the piecewise-linear spring model as proposed by Luo and Hanagud [13]. The spring stiffness is then set to be equal to zero (0) for the *free mode* and infinity (*∞*) for the *constrained mode*.

**Figure 2:**
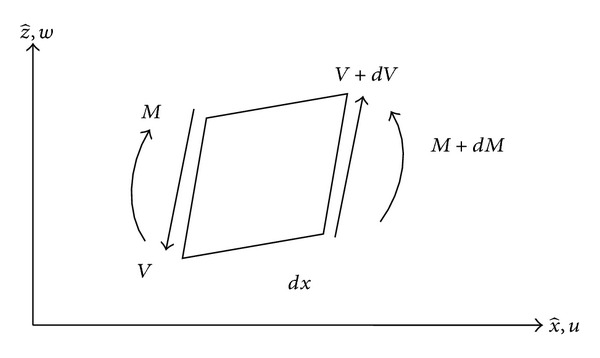
Sign convention for bending moment *M* and shear force *V*.

The kinetic energy *T* and the strain energy *U* (under the sign convention for bending moment and shear force shown in Figure 2) of the vibrating delaminated beam can be written as

(1) T=∑i=1412∫0Li{miwi,t2+miri2ψi,t2+miui,t2}dx^,U=∑i=1412∫0Li{EIiψi,x^2+ksAiG(wi,x^−ψi)2+EAiui,x^2}dx^     +12∫0L2k(w2−w3)2dx^,

where ($w_{i,\hat{x}}-\psi_{i},\;i=1,2,3,4$) represents the shear angle, $w_{i}(\hat{x},t)$, $\psi_{i}\left(\hat{x},t\right)$, and $u_{i}(\hat{x},t)$ are the transverse displacement, the cross-section rotation due to the bending moment, and the axial displacement of the *i*th subbeam, $\hat{x}$ is the axial coordinate of the subbeam whose origin is located at the left boundary of each subbeam, *r*_*i*_ is the radius of gyration ($=\sqrt{I_{i}/A_{i}}$), *k*_*s*_ is the beam cross-sectional shape factor, and *k* is the spring stiffness. It should be mentioned that in all above relations, the symbol “,” used as a subscript stands for the differentiation with respect to any variable followed after it. The above relations for the kinetic and potential energies will be used in Section 3 to form the functional.

## 3. Analytical Solution

In the present work, series solutions in conjunction with the Lagrange multipliers are used to study the free vibration characteristics of the delaminated beam. The main advantage of the Lagrange multiplier technique is that the choice of the assumed displacement functions is easy because they do not have to satisfy the boundary conditions of the problem. The simple Legendre polynomials are chosen as displacement functions, and this simplifies the problem further in that orthogonality properties of these polynomials lead to simple energy expression.

Harmonic solutions for the variables $w_{i}(\hat{x},t)$, $\psi_{i}(\hat{x},t)$ and $u_{i}\left(\hat{x},t\right)$, are assumed as

(2) wi(x^,t)=Wi(x^)eiωt,ψi(x^,t)=Ψi(x^)eiωtui(x^,t)=Ui(x^)eiωt,  (i=1,  2,  3,  4)

in which $W_{i}\left(\hat{x}\right)$, $\Psi_{i}\left(\hat{x}\right)$, and $U_{i}(\hat{x})$ are the displacement functions of the *i*th subbeam and *ω* is the circular frequency. As mentioned above, the displacement functions can be expressed in terms of the simple Legendre polynomials and are given by

(3) $$\begin{array}{c} W_{i}\left(x\right)=\sum_{m=0}^{n_{t}}W_{im}P_{m}\left(x\right),\;\;\;\;\\ \Psi_{i}\left(x\right)=\sum_{m=0}^{n_{t}}\Psi_{im}P_{m}\left(x\right),\;\\ U_{i}\left(x\right)=\sum_{m=0}^{n_{t}}U_{im}P_{m}\left(x\right). \end{array}$$

Here, *P*_*m*_ is the simple Legendre polynomial of degree *m* and the axial coordinate $\hat{x}$ is transformed to the interval −1 ≤ *x* ≤ 1 by letting $x=\left(\hat{x}-L_{i}/2\right)/\left(L_{i}/2\right)$.

We have six boundary conditions (BCs) for subbeams 1 and 4, that is, three boundary conditions for each subbeam. These six boundary conditions which are not satisfied by the assumed series are imposed as constraints. For common boundary conditions, these constraints can be written as follows.

Clamped-Clamped Beam (C-C):

(4a) W1(−1)=0,  Ψ1(−1)=0,  U1(−1)=0,W4(1)=0,   Ψ4(1)=0,[Immovable (I:C-C):U4(1)=0 or  Movable (M:C-C):U4′(1)=0].

Clamped-Hinged Beam (C-H):

(4b) W1(−1)=0,  Ψ1(−1)=0,  U1(−1)=0,W4(1)=0,  Ψ4′(1)=0,[Immovable (I:C-H):U4(1)=0 or  Movable (M:C-H):U4′(1)=0].

Hinged-Hinged Beam (H-H):

(4c) W1(−1)=0,  Ψ1′(−1)=0,  U1(−1)=0,W4(1)=0,  Ψ4′(1)=0,[Immovable (I:H-H):U4(1)=0   or  Movable (M:H-H):U4′(1)=0].

Clamped-Free Beam (C-F):

(4d) W1(−1)=0,  Ψ1(−1)=0,[Immovable (I:C-F):U1(−1)=0 or  Movable (M:C-F):U1′(−1)=0]  2LW4′(1)−Ψ4(1)=0,  Ψ4′(1)=0,  U4′(1)=0

in which prime denotes differentiation with respect to *x*. Furthermore, we have 18 continuity and compatibility conditions (C.C.s) at delamination boundaries as follows.

At the left end of subbeams 2 and 3:

(5) W1(1)=W2(−1), W1(1)=W3(−1), Ψ1(1)=Ψ2(−1),Ψ1(1)=Ψ3(−1),  U1(1)−H22L1W1′(1)=U2(−1),U1(1)+H32L1W1′(1)=U3(−1),M1(1)−M2(−1)−M3(−1)+H2N2(−1)−H3N3(−1)=0,V1(1)=V2(−1)+V3(−1),  N1(1)=N2(−1)+N3(−1),

at the right end of subbeams 2 and 3:

(6) W2(1)=W4(−1), W3(1)=W4(−1), Ψ2(1)=Ψ4(−1),Ψ3(1)=Ψ4(−1),  U4(−1)−H22L4W4′(−1)=U2(1),U4(−1)+H32L4W4′(−1)=U3(1),M4(1)−M2(1)−M3(1)+H2N2(1)−H3N3(1)=0,V4(−1)=V2(1)+V3(1),  N4(−1)=N2(1)+N3(1),

where *M*_*i*_,  *V*_*i*_, and *N*_*i*_ are the bending moment, shear, and axial forces, respectively. Boundary, continuity, and compatibility conditions in terms of assumed series are presented in the appendix.

A variational principle is formulated based on the kinetic and strain energies by a procedure similar to the one followed by Washizu [14]. This variational principle along with the constraint conditions is used to solve the vibration problem. The functional to be extremized is given by the expression

(7) F=U+T−∑i=16αi(B.C.s)−∑i=19βi(C.C.s  at  x=L1) −∑i=19γi(C.C.s  at  x=L1+L2),

where  *α*_*i*_  (*i* = 1,…, 6), *β*_*i*_ and *γ*_*i*_  (*i* = 1,2,…, 9) are the Lagrange multipliers. Substituting the assumed series for *W*_*i*_(*x*), Ψ_*i*_(*x*),  and  *U*_*i*_(*x*)  (*i* = 1,2, 3,4) in (7) and simplifying yields

(8) F=∑i=14[EIiLi∫−11∑m=1ntΨim∑k1=0⌊(m−1)/2⌋(2m−4k1−1)Pm−2k1−1(x)   ×∑n=1ntΨin∑k2=0⌊(n−1)/2⌋(2n−4k2−1)Pn−2k2−1(x)dx   +kAiGLi4∑m=0nt22m+1Ψim2+kAiGLi   ×∫−11∑m=1ntWim∑k1=0⌊(m−1)/2⌋(2m−4k1−1)Pm−2k1−1(x)   ×∑n=1ntWin∑k2=0⌊(n−1)/2⌋(2n−4k2−1)Pn−2k2−1(x)dx−kAiG   ×∫−11∑m=0ntΨimPm(x)∑n=1ntWin∑k1=0⌊(n−1)/2⌋(2n−4k1−1)×Pn−2k1−1(x)dx   +EAiLi∫−11∑m=1ntUim∑k1=0⌊(m−1)/2⌋(2m−4k1−1)Pm−2k1−1(x)   ×∑n=1ntUin∑k2=0⌊(n−1)/2⌋(2n−4k2−1)Pn−2k2−1(x)dx   −miω2Li4∑m=0nt22m+1(Wim2+ri2Ψim2+Uim2)]+kL2b4∑m=0nt22m+1(W2m2+W3m2−2W2mW3m)−∑i=16αi(B.C.s)−∑i=19βi(C.E.s  at  x=L1)−∑i=19γi(C.E.s  at  x=L1+L2).

In calculating the functional *F*, we have used the following properties of Legendre polynomial [15]:

(9) $$\begin{array}{c} P_{n}^{\prime }\left(x\right)=\sum_{k=0}^{\left\lfloor \left(n-1\right)/2\right\rfloor }\left(2n-4k-1\right)P_{n-2k-1}\left(x\right)\;\left(n\geq 1\right). \end{array}$$

The necessary extremizing conditions are given by

(10) ∂F∂Wim=∂F∂Ψim=∂F∂Uim=0,(m=0,1,2,…), (i=1,2,3,4).

Using (10) in conjunction with (8) results in a system of linear algebraic equations which, in matrix form, can be written as

(11) $$\begin{array}{c} \left[A\right]{\left\{q_{1},q_{2},q_{3},q_{4}\right\}}^{T}=\left[B\right], \end{array}$$

in which the right-hand side of (11) consists of Lagrange multipliers and

(12) $$\begin{array}{c} q_{i}=\left\{W_{i0},W_{i1},\ldots ,W_{im},\Psi_{i0},\Psi_{i1},\ldots ,\Psi_{im},U_{i0},U_{i1},\ldots ,U_{im}\right\}. \end{array}$$

Solving (11) for *W*_*im*_, Ψ_*im*_  and  *U*_*im*_  (*i* = 1,2, 3,4) and substituting into (4a), (4b), and (4c)–(6) results in a system of homogeneous linear algebraic equations with the Lagrange multipliers as unknowns. The system of equations is given by

(13) $$\begin{array}{c} \left[C\right]{\left\{\alpha_{1},\alpha_{2},\ldots ,\alpha_{6},\beta_{1},\beta_{2},\ldots ,\beta_{9},\gamma_{1},\gamma_{2},\ldots ,\gamma_{9}\right\}}^{T}={\left\{0\right\}}^{T}. \end{array}$$

The natural frequencies and corresponding mode shapes of beams can be calculated using (11) and (13). In calculating the natural frequency, the determinant of the coefficient matrix in (13) is computed for various values of frequency starting from a near zero value. Zero crossing of the determinant is identified and the corresponding value of frequency is the natural frequency of the beam in question.

## 4. Results and Discussion

In order to demonstrate the high accuracy of the present method, the results for the delaminated beams are compared with the available exact solution. A variety of results are presented for Timoshenko beams with different boundary conditions and single delamination for which the exact solutions do not exist. For all the problems, the width of the beam is taken as unity and the shear correction factor and Poisson ratio are taken to be 5/6 and 0.3, respectively.

In order to present the obtained results in a standard way, we use the following nondimensional parameters ${\bar{L}}_{1}$, ${\bar{L}}_{2}$, and ${\bar{H}}_{2}$ which represent the spanwise location of the delamination, its length, and its thickness, respectively:

(14) $$\begin{array}{c} {\bar{L}}_{1}=\frac{L_{1}}{L},\;\;\;{\bar{L}}_{2}=\frac{L_{2}}{L},\;\;{\bar{H}}_{2}=\frac{H_{2}}{H_{1}}. \end{array}$$

Also, for all the cases, the natural frequencies are presented using the dimensionless form ($\Omega =\omega L^{2}\sqrt{m_{1}/EI_{1}}$).

### 4.1. Natural Frequencies

**Figure 3:**
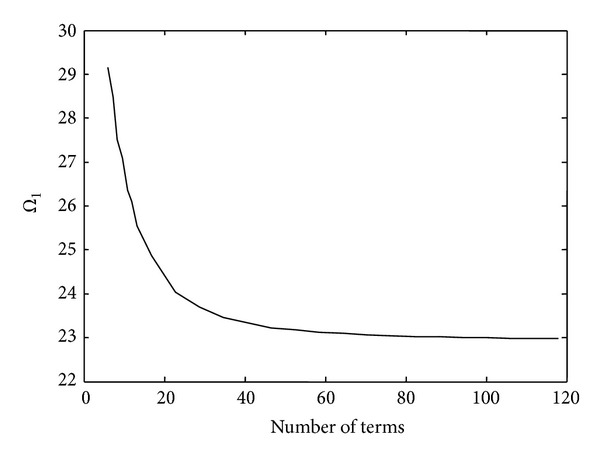
Convergence study for a delaminated Timoshenko beam with I:C-C B.C.s (${\bar{L}}_{1}=0.3$, ${\bar{L}}_{2}=0.4$, ${\bar{H}}_{2}=0.5$, and *L*/*H*_1_ = 10).

In Figure 3, a convergence study in order to determine the number of terms required to obtain convergent solutions is shown. It is clear that converged solutions are obtained if the number of terms taken is 100 or more. All the problems considered hereafter are based on the first 100 terms of the series.

A cantilever beam containing a delamination along the midplane of the beam has been considered. The beam is considered to be fixed at the left end. The fundamental frequencies of the beam corresponding to delamination lengths of 0.1 and 0.3 at various locations are compared with analytical solution based on the Euler-Bernoulli beam theory [1] in Table 1. It can be seen that the natural frequency obtained from Timoshenko beam model yields the same value provided that the beam's slenderness ratio is at least 100.

For further demonstration of the proficiency of the present method, more numerical calculations for delaminated beams are performed. In Tables 2 and 3, the dimensionless fundamental frequencies of an I:C-C slender beam (*L*/*H*_1_ = 200) with central and midplane delamination having various lengths are compared with the analytical results of Wang et al. [1] and Della et al. [6] and FEM results of Lee [16].  Table 2 shows excellent agreement between the present results and analytical and FEM results.

The variations of fundamental frequencies for a beam having a delamination of various lengths at different spanwise locations have been computed and are presented in Table 3. From the results, it can be seen that the fundamental frequency decreases as the delamination length increases, as anticipated. The fundamental frequency is generally unaffected by the location of the split region for very short delaminations. The frequency decreases when the delamination location is moved from the central location of the beam toward the supporting end, and the change of frequency is noticeable, particularly for longer delaminations.

Since the conventional beam theories cannot involve the effect of Poisson ratio, it is rather interesting to take a deep insight into it using the present approach.  Table 4 gives the variation of the fundamental frequency parameter (*Ω*) of I:C-F beams with the Poisson ratio based on the *free* and *constrained mode models*. It is shown that the natural frequency decreases gradually with the increasing of Poisson ratio. We can see that the natural frequencies for *ν* = 0.5 have a deviation from that those for *ν* = 0.1. From this point of view, the Poisson ratio is of great significance in structural design especially for composite material beams.

### 4.2. Mode Shapes

**Figure 4:**
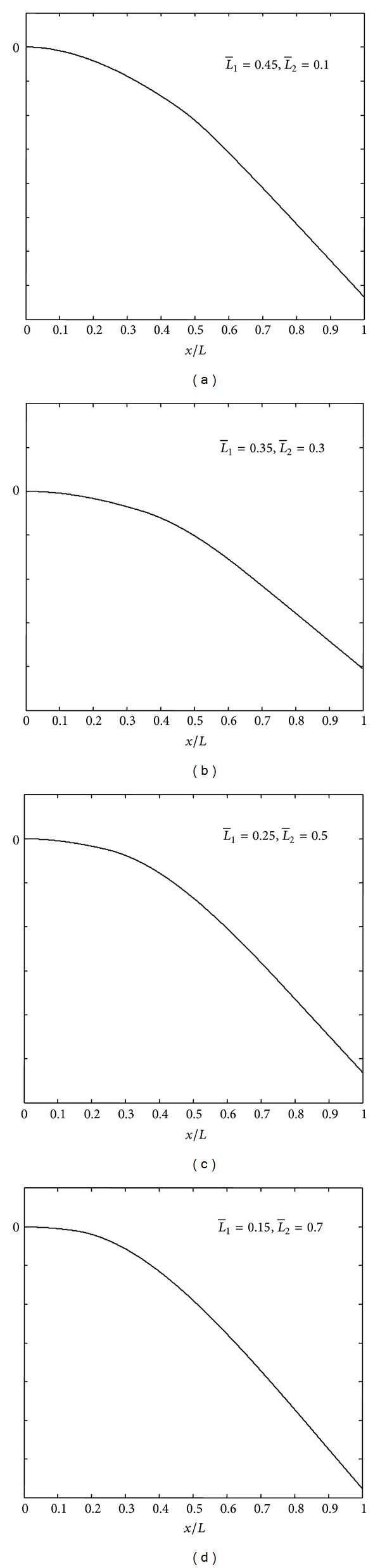
Mode shapes of the delaminated beam with (${\bar{H}}_{2}=0.5$).

**Figure 5:**
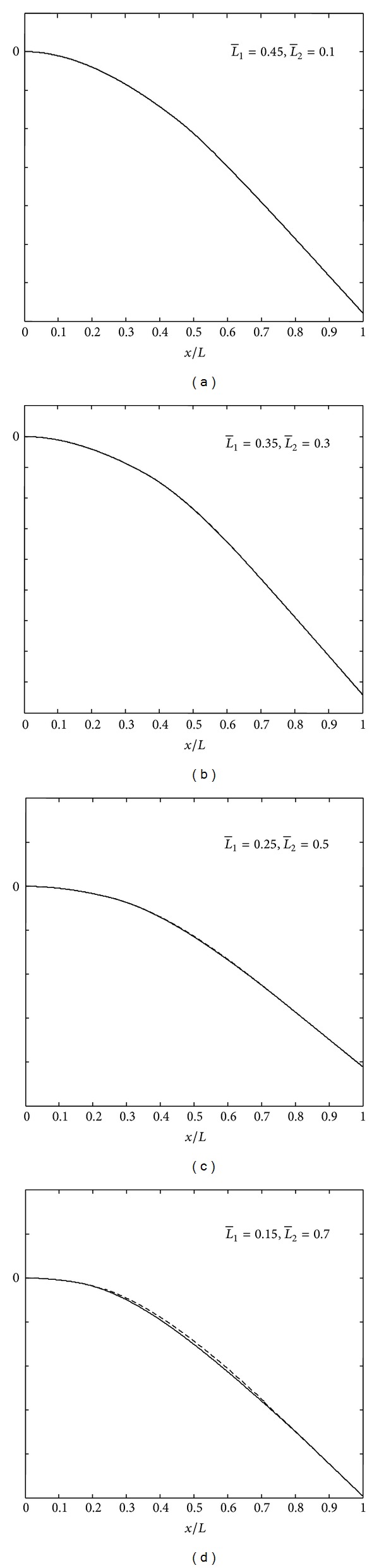
Mode shapes of the delaminated beam with (${\bar{H}}_{2}=0.3$).

**Figure 6:**
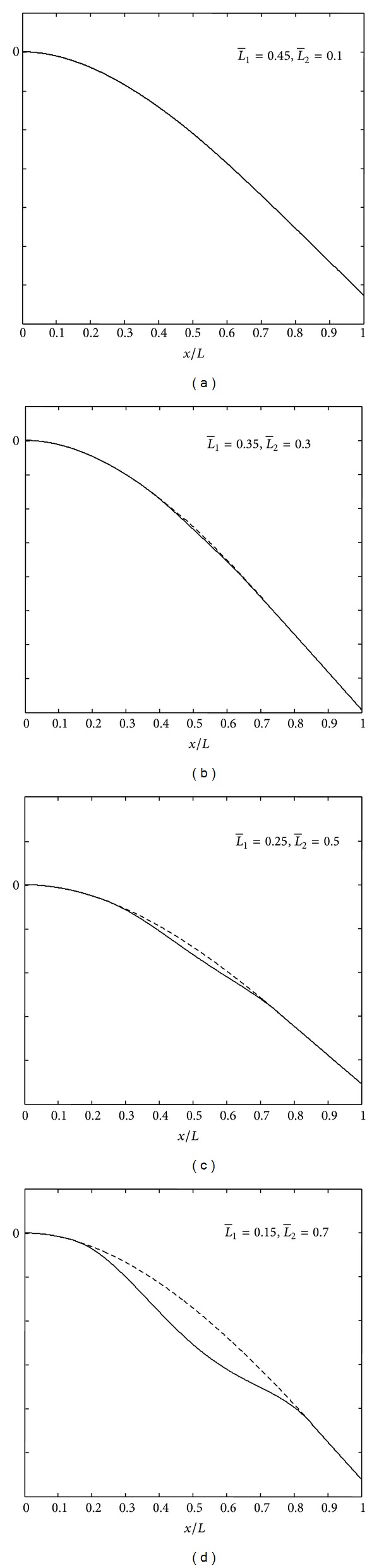
Mode shapes of the delaminated beam with (${\bar{H}}_{2}=0.1$).

In Figures 4, 5, and 6 are shown the mode shapes of the thick beams (*L*/*H*_1_ = 10) with different delamination location and size at *k* = 0 (*free mode*) and M:C-F boundary conditions. From these figures, we can see that first vibration modes do not show any opening in the cases of short delaminations and closeness to the midplane of the beam, while in cases of long delaminations and closeness to the beam surface, we can clearly see the delamination opening modes. By referring to Tables 1, 2, 3, and 4, it is clear that for modes where there is no opening in the delamination region, frequencies predicted by the free (*k* = 0) and constrained (*k* = *∞*) modes yield the same value or are very close to each other. This is reasonable since if there is no opening in the delamination region, the *free mode* and *constrained* model are essentially the same.

## 5. Conclusions

The free vibration of the Timoshenko beams is investigated using an assumed series solution in conjunction with Lagrange multipliers. It is observed that the present method is a computationally efficient tool in predicting the natural frequencies of the beams. This method is particularly attractive because of the ease with which one can choose the generalized displacement functions. This fact is demonstrated by choosing Legendre polynomials, whose orthogonal properties of which simplify energy expressions considerably. The natural frequencies of the Timoshenko beam obtained by this method compare extremely well with the available exact solution.

## Figures and Tables

**Table 1:** Nondimensional fundamental frequency (Ω_1_) of an I:C-F beam with a midplane delamination.

${\bar{L}}_{2}$	${\bar{L}}_{1}$	Analytical [1]	*L*/*H*_1_						
			10	25	50	100	200	250	300
0.1	0.2	3.5126	2.8338	2.8542	2.8541	2.9968	3.1531	3.3015	3.4118
	0.4	3.5128	3.1922	3.2163	3.2308	3.2749	3.3769	3.4964	3.5101
	0.6	3.5137	3.4169	3.4425	3.4491	3.4602	3.4861	3.5013	3.5107
	0.8	3.5148	3.4818	3.5078	3.5118	3.5122	3.5122	3.5122	3.5122

0.3	0.2	3.438	2.4421	2.4816	2.5701	2.8298	3.2839	3.4212	3.4311
	0.4	3.459	3.0117	3.0513	3.1040	3.2377	3.4170	3.4453	3.4472
	0.6	3.489	3.3842	3.4198	3.4324	3.4561	3.4825	3.4850	3.4851

**Table 2:** First three dimensionless natural frequencies of the beam with a central and midplane delamination.

	Method	${\bar{L}}_{2}$									
		0.001	0.1	0.2	0.3	0.4	0.5	0.6	0.7	0.8	0.9
*Ω*_1_	Present	22.39	21.48	22.20	21.34	21.69	21.36	19.91	17.68	15.25	13.06
	Analytical [6]	22.37	22.37	22.36	22.24	21.83	20.89	19.30	17.23	15.05	13.00
	Analytical [1]	22.39	22.37	22.35	22.23	21.83	20.88	19.29	17.23	15.05	13.00
	FEM [16]	22.36	22.36	22.35	22.23	21.82	20.88	19.28	17.22	15.05	12.99

*Ω*_2_	Present	61.66	60.79	55.98	48.99	43.93	41.52	40.98	40.81	39.06	35.39
	Analytical [6]	61.67	60.81	56.00	49.00	43.89	41.52	41.04	40.82	39.07	35.39
	Analytical [1]	61.67	60.76	55.97	49.00	43.87	41.45	40.93	40.72	39.01	35.38
	FEM [16]	61.61	60.74	55.95	48.97	43.86	41.50	41.01	40.80	39.04	35.38

*Ω*_3_	Present	120.75	115.13	118.02	107.22	93.58	82.36	78.28	78.61	76.57	69.50
	Analytical [6]	120.90	120.83	118.87	109.16	93.59	82.29	77.69	77.18	75.43	69.19
	Analytical [1]	120.91	120.81	118.76	109.04	93.57	82.29	77.64	77.05	75.33	69.17
	FEM [16]	120.68	120.62	118.69	109.03	93.51	82.23	77.64	77.12	75.39	69.16

**Table 3:** Dimensionless fundamental frequencies of the I:C-C beam with a central and midplane delamination.

${\bar{L}}_{2}$	Method	${\bar{L}}_{1}$			
		0.1	0.2	0.3	0.4
0.1	Present	21.75	22.26	21.49	20.71
	Analytical [1]	22.15	22.21	22.30	22.36

0.2	Present	20.97	21.23	20.97	20.71
	Analytical [1]	21.00	21.53	22.10	22.35

0.3	Present	19.16	20.45	21.23	21.17
	Analytical [1]	19.34	20.78	22.02	22.02

0.4	Present	18.13	20.45	21.74	20.41
	Analytical [1]	18.18	20.48	21.83	20.48

**Table 4:** Effect of Poisson ratio on the fundamental frequency parameter (Ω) of I:C-F beams with central and midplane delamination (*L*/*H*_1_ = 10).

${\bar{H}}_{2}$	*k*	*ν*				
		0.1	0.2	0.3	0.4	0.5
0.5	0	2.6991	2.6975	2.6961	2.6947	2.6933
	∞	2.6991	2.6975	2.6961	2.6947	2.6933

0.2	0	3.1299	3.1149	3.0933	3.0617	3.0172
	∞	3.1303	3.1153	3.0936	3.0620	3.0173

## Appendix

By substituting (3) in (4a), (4b), and (4c) the B.C.s can be written as

Clamped-Clamped Beam (C-C):

(A.1) ∑m=0nt(−1)mW1m=0,  ∑m=0nt(−1)mΨ1m=0,  ∑m=0nt(−1)mU1m=0∑m=0ntW4m=0,  ∑m=0ntΨ4m=0,[I:C-C:∑m=0nt(−1)mU4m=0     or  M:C-C:∑m=1ntU4m∑k1=0⌊(m−1)/2⌋(2m−4k1−1)=0].

Clamped-Hinged Beam (C-H):

(A.2) ∑m=0nt(−1)mW1m=0,  ∑m=0nt(−1)mΨ1m=0,  ∑m=0nt(−1)mU1m=0,∑m=0ntW4m=0,  ∑m=1ntΨ4m∑k1=0⌊(m−1)/2⌋(2m−4k1−1)=0,[I:C-H:∑m=0nt(−1)mU4m=0 or  M:C-H:∑m=1ntU4m∑k1=0⌊(m−1)/2⌋(2m−4k1−1)=0].    

Hinged-Hinged Beam (H-H):

(A.3) ∑m=0nt(−1)mW1m=0,  ∑m=1ntΨ1m∑k1=0⌊(m−1)/2⌋(−1)m−2k1−1(2m−4k1−1)=0,∑m=0nt(−1)mU1m=0,  ∑m=0ntW4m=0,∑m=1ntΨ4m∑k1=0⌊(m−1)/2⌋(2m−4k1−1)=0,[I:H-H:∑m=0nt(−1)mU4m=0 or    M:H-H:∑m=1ntU4m∑k1=0⌊(m−1)/2⌋(2m−4k1−1)=0].

Clamped-Free Beam (C-F):

(5) ∑m=0nt(−1)mW1m=0,  ∑m=0nt(−1)mΨ1m=0,[I:C-F:∑m=0nt(−1)mU1m=0   or  M:C-F: ∑m=1ntU1m∑k1=0⌊(m−1)/2⌋(2m−4k1−1)×(−1)m−2k1−1=0]2L∑m=1ntW4m∑k1=0⌊(m−1)/2⌋(2m−4k1−1)   −∑m=0ntΨ4m=0,  ∑m=1ntΨ4m∑k1=0⌊(m−1)/2⌋(2m−4k1−1)=0,∑m=1ntU4m∑k1=0⌊(m−1)/2⌋(2m−4k1−1)=0.

Also, by substituting (3) in (5) and (6), the C.C.s can be expressed as follows.

At the left end of subbeams 2 and 3:

(A.5a) ∑m=0nt[W1m−(−1)mW2m]=0,  ∑m=0nt[W1m−(−1)mW3m]=0,∑m=0nt[Ψ1m−(−1)mΨ2m]=0,  ∑m=0nt[Ψ1m−(−1)mΨ3m]=0,∑m=0nt[U1m−(−1)mU2m]−2H2L1∑m=1ntW1m∑k1=0⌊(m−1)/2⌋(2m−4k1−1)=0,∑m=0nt[U1m−(−1)mU3m]+2H3L1∑m=1⌊(m−1)/2⌋W1m∑k1=0nt(2m−4k1−1)=0,I1L1∑m=1ntΨ1m∑k1=0⌊(m−1)/2⌋(2m−4k1−1) +∑m=1nt[−I2LdΨ2m−I3LdΨ3m+A2H2LdU2m−A3H3LdU3m] ×∑k1=0⌊(m−1)/2⌋(2m−4k1−1)=0,∑m=0nt[−A1Ψ1m+A2Ψ2m(−1)m+A3Ψ3m(−1)m]+2A1L1∑m=1ntW1m ×∑k1=0⌊(m−1)/2⌋(2m−4k1−1) +∑m=1nt[−2A2LdW2m−2A3LdW3m]∑k1=0⌊(m−1)/2⌋(2m−4k1−1)=0,A1L1∑m=1ntU1m∑k1=0⌊(m−1)/2⌋(2m−4k1−1) +∑m=1nt[−A2LdU2m−A3LdU3m]∑k1=0⌊(m−1)/2⌋(2m−4k1−1)(−1)m−2k1−1=0,

at the right end of subbeams 2 and 3:

(A.6) ∑m=0nt[(−1)mW4m−W2m]=0,  ∑m=0nt[(−1)mW4m−W3m]=0,∑m=0nt[(−1)mΨ4m−Ψ2m]=0,  ∑m=0nt[(−1)mΨ4m−Ψ3m]=0,∑m=0nt[(−1)mU4m−U2m]−2H2L4∑m=1ntW4m ×∑k1=0⌊(m−1)/2⌋(2m−4k1−1)(−1)m−2k1−1=0,∑m=0nt[(−1)mU4m−U3m]+2H3L4∑m=1⌊(m−1)/2⌋W4m ×∑k1=0nt(2m−4k1−1)(−1)m−2k1−1=0,I1L4∑m=1ntΨ4m∑k1=0⌊(m−1)/2⌋(2m−4k1−1)(−1)m−2k1−1 +∑m=1nt[−I2LdΨ2m−I3LdΨ3m+A2H2LdU2m−A3H3LdU3m] ×∑k1=0⌊(m−1)/2⌋(2m−4k1−1)=0,∑m=0nt[−A1(−1)mΨ1m+A2Ψ2m+A3Ψ3m] +2A1L4∑m=1ntW4m∑k1=0⌊(m−1)/2⌋(2m−4k1−1)(−1)m−2k1−1 +∑m=1nt[−2A2LdW2m−2A3LdW3m]∑k1=0⌊(m−1)/2⌋(2m−4k1−1)=0,A1L4∑m=1ntU4m∑k1=0⌊(m−1)/2⌋(2m−4k1−1)(−1)m−2k1−1 +∑m=1nt[−A2LdU2m−A3LdU3m]∑k1=0⌊(m−1)/2⌋(2m−4k1−1)=0.
